# Bis(5,6-dicarboxy­benzimidazolium) sulfate monohydrate

**DOI:** 10.1107/S1600536809018182

**Published:** 2009-05-20

**Authors:** Yue Cui, Qian Gao, Chao-Yan Zhang, Ya-Bo Xie

**Affiliations:** a College of Environmental and Energy Engineering, Beijing University of Technology, Beijing 100124, People’s Republic of China

## Abstract

In the title compound, 2C_9_H_7_N_2_O_4_^+^·SO_4_^2−^·H_2_O, the sulfate S atom and the water O atom reside on a crystallographic twofold axis. In the crystal, the component species are linked by N—H⋯O, O—H⋯O and C—H⋯O hydrogen bonds, forming a three-dimensional network structure. An intramol­ecular O—H⋯O link is seen in the cation.

## Related literature

For a related structure that contains a benzimidazole mol­ecule, see: Gao *et al.* (2008 ▶). For the pharmacokinetics of an anti­allergic benzimidazole derivative, see: Sakai *et al.* (1989 ▶). For the synthesis and chemoluminescence of an amino drivative, see: White & Matsuo (1967 ▶).
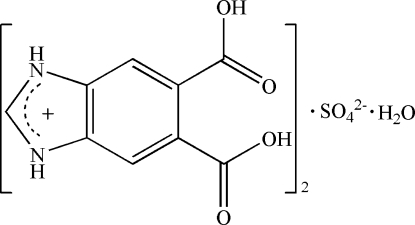


## Experimental

### 

#### Crystal data

2C_9_H_7_N_2_O_4_^+^·SO_4_^2−^·H_2_O
*M* *_r_* = 528.41
Orthorhombic, 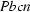

*a* = 14.691 (3) Å
*b* = 7.7968 (17) Å
*c* = 17.983 (4) Å
*V* = 2059.8 (8) Å^3^
*Z* = 4
Mo *K*α radiation
μ = 0.24 mm^−1^
*T* = 296 K
0.12 × 0.11 × 0.10 mm

#### Data collection

Bruker SMART CCD area-detector diffractometer
Absorption correction: multi-scan (*SADABS*; Bruker, 1998 ▶) *T*_min_ = 0.971, *T*_max_ = 0.976
11525 measured reflections
2413 independent reflections
1994 reflections with *I* > 2σ(*I*)
*R*_int_ = 0.060

#### Refinement

*R*[*F* ^2^ > 2σ(*F* ^2^)] = 0.048
*wR*(*F* ^2^) = 0.138
*S* = 1.00
2413 reflections
168 parameters
H atoms treated by a mixture of independent and constrained refinement
Δρ_max_ = 0.46 e Å^−3^
Δρ_min_ = −0.40 e Å^−3^

### 

Data collection: *SMART* (Bruker, 1998 ▶); cell refinement: *SAINT* (Bruker, 1998 ▶); data reduction: *SAINT*; program(s) used to solve structure: *SHELXS97* (Sheldrick, 2008 ▶); program(s) used to refine structure: *SHELXL97* (Sheldrick, 2008 ▶); molecular graphics: *SHELXTL* (Sheldrick, 2008 ▶); software used to prepare material for publication: *SHELXTL*.

## Supplementary Material

Crystal structure: contains datablocks global, I. DOI: 10.1107/S1600536809018182/si2172sup1.cif

Structure factors: contains datablocks I. DOI: 10.1107/S1600536809018182/si2172Isup2.hkl

Additional supplementary materials:  crystallographic information; 3D view; checkCIF report

## Figures and Tables

**Table 1:** Hydrogen-bond geometry (Å, °)

*D*—H⋯*A*	*D*—H	H⋯*A*	*D*⋯*A*	*D*—H⋯*A*
N2—H22⋯O1*W*^ii^	0.90	1.96	2.8365 (10)	163
N1—H25⋯O11^iii^	0.88	1.82	2.6931 (12)	175
C5—H5*A*⋯O2^v^	0.93	2.20	3.098 (3)	162
O1—H28⋯O9^iv^	0.85	1.84	2.6616 (11)	161
O4—H21⋯O11	0.85	1.79	2.6330 (11)	169
O1*W*—H1*WA*⋯O3^i^	0.96 (6)	2.26 (5)	2.9012 (11)	123.6
O1*W*—H1*WA*⋯O11^i^	0.96 (6)	2.47 (6)	3.1575 (16)	128.4

Symmetry codes: (i) 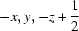; (ii) 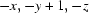; (iii) 
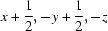
; (iv) 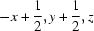; (v) 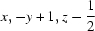.

**Fig. 1.**
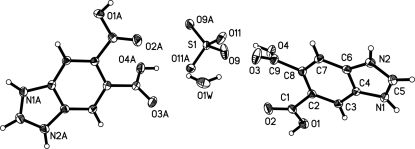
A view of the molecular structure of the title compound with displacement ellipsoids drawn at the 30% probability level for non-hydrogen atoms. Symmetry related atoms labelled A have the symmetry code A = -x, y, 1/2 - z.

## supplementary crystallographic information

### Comment

Benzimidazole and related heterocyclic compounds have been extensively investigated because of their pharmacological activities (Sakai *et al.*, 1989) and the application as intermediate for the synthesis of chemiluminescent compound (White & Matsuo, 1967). Otherwise, this kind of compounds is one of the most prevalent ligands in the field of coordination chemistry (Gao *et al.*, 2008). Herein, we report the crystal structure of the title compound (Fig. 1), Bis(1*H*-benzimidazolium-5,6-dicarboxyl) sulfate monohydrate.

The title compound consists of two 1*H*-benzimidazole-5,6-dicarboxylic acid cations, one sulfate dianion and one water molecule. The sulfate S atom and the water O atom reside on crystallographic twofold axis. As one imine N atom on the benzimidazolium ring is protonated, there exsist positive charge in the ring (Scheme 1). The cations, dianions and water molecules are linked through a combination of intermolecular N—H···O, O—H···O and C—H···O hydrogen bonds (Table 1) to form a three-dimensional network structure.

### Experimental

A solution containing a 2:1 molar ratio of ZnSO_4_ and 1*H*-benzoimidazole-5,6-dicarboxylate in water was sealed in a 25 ml teflon reactor and kept at 393K for 3 days. Then the mixture was filtered and the filtrate was allowed to stand at room temperature. Colorless block crystals suitable for the X-ray investigation were collected.

### Refinement

The water H atoms were located in a difference Fourier map and freely refined. The N-bound H atoms were located in a difference Fourier map and fixed during the refinement with *U*_iso_(H) = 1.2*U*_eq_(N). The C-bound H atoms were positioned geometrically (C—H = 0.93 Å) and refined as riding with *U*_iso(H)_ = 1.2*U*_eq_(C).

### Crystal data

**Table:**

2C_9_H_7_N_2_O_4_^+^·SO_4_^2^^−^·H_2_O	*F*(000) = 1088
*M**_r_* = 528.41	*D*_x_ = 1.704 Mg m^−^^3^
Orthorhombic, *P**b**c**n*	Mo *K*α radiation, λ = 0.71073 Å
Hall symbol: -P 2n 2ab	Cell parameters from 2413 reflections
*a* = 14.691 (3) Å	θ = 2.3–25.0°
*b* = 7.7968 (17) Å	µ = 0.24 mm^−^^1^
*c* = 17.983 (4) Å	*T* = 296 K
*V* = 2059.8 (8) Å^3^	Block, colorless
*Z* = 4	0.12 × 0.11 × 0.10 mm

### Data collection

**Table:**

Bruker SMART CCD area-detector diffractometer	2413 independent reflections
Radiation source: fine-focus sealed tube	1994 reflections with *I* > 2σ(*I*)
graphite	*R*_int_ = 0.060
φ and ω scans	θ_max_ = 27.8°, θ_min_ = 2.3°
Absorption correction: multi-scan (*SADABS*; Bruker, 1998)	*h* = −17→16
*T*_min_ = 0.971, *T*_max_ = 0.976	*k* = −9→9
11525 measured reflections	*l* = −21→17

### Refinement

**Table:**

Refinement on *F*^2^	Primary atom site location: structure-invariant direct methods
Least-squares matrix: full	Secondary atom site location: difference Fourier map
*R*[*F*^2^ > 2σ(*F*^2^)] = 0.048	Hydrogen site location: inferred from neighbouring sites
*wR*(*F*^2^) = 0.138	H atoms treated by a mixture of independent and constrained refinement
*S* = 1.00	*w* = 1/[σ^2^(*F*_o_^2^) + (0.0847*P*)^2^ + 0.8915*P*] where *P* = (*F*_o_^2^ + 2*F*_c_^2^)/3
2413 reflections	(Δ/σ)_max_ < 0.001
168 parameters	Δρ_max_ = 0.46 e Å^−^^3^
0 restraints	Δρ_min_ = −0.40 e Å^−^^3^

### Special details

**Table:**

Geometry. All e.s.d.'s (except the e.s.d. in the dihedral angle between two l.s. planes) are estimated using the full covariance matrix. The cell e.s.d.'s are taken into account individually in the estimation of e.s.d.'s in distances, angles and torsion angles; correlations between e.s.d.'s in cell parameters are only used when they are defined by crystal symmetry. An approximate (isotropic) treatment of cell e.s.d.'s is used for estimating e.s.d.'s involving l.s. planes.
Refinement. Refinement of *F*^2^ against ALL reflections. The weighted *R*-factor *wR* and goodness of fit *S* are based on *F*^2^, conventional *R*-factors *R* are based on *F*, with *F* set to zero for negative *F*^2^. The threshold expression of *F*^2^ > σ(*F*^2^) is used only for calculating *R*-factors(gt) *etc*. and is not relevant to the choice of reflections for refinement. *R*-factors based on *F*^2^ are statistically about twice as large as those based on *F*, and *R*- factors based on ALL data will be even larger.

### Fractional atomic coordinates and isotropic or equivalent isotropic displacement parameters (Å^2^)

**Table:**

	*x*	*y*	*z*	*U*_iso_*/*U*_eq_	
O1	0.35417 (10)	0.1890 (2)	0.09502 (8)	0.0418 (4)	
H28	0.3685	0.1903	0.1408	0.050*	
O2	0.22201 (11)	0.2483 (3)	0.15019 (8)	0.0553 (5)	
O3	0.00994 (12)	0.3035 (2)	0.08983 (10)	0.0586 (5)	
O4	0.08286 (11)	0.05673 (19)	0.07644 (9)	0.0474 (4)	
H21	0.0465	0.0172	0.1089	0.057*	
N1	0.29633 (11)	0.4817 (2)	−0.16017 (8)	0.0344 (4)	
H25	0.3535	0.4982	−0.1718	0.041*	
N2	0.15053 (11)	0.5073 (2)	−0.17520 (9)	0.0358 (4)	
H22	0.0969	0.5355	−0.1962	0.043*	
C1	0.26786 (14)	0.2354 (2)	0.09487 (10)	0.0339 (4)	
C2	0.23266 (12)	0.2806 (2)	0.01950 (9)	0.0280 (4)	
C3	0.29283 (11)	0.3365 (2)	−0.03408 (10)	0.0293 (4)	
H3A	0.3554	0.3271	−0.0276	0.035*	
C4	0.25595 (12)	0.4077 (2)	−0.09819 (9)	0.0282 (4)	
C5	0.23151 (14)	0.5390 (3)	−0.20452 (11)	0.0381 (4)	
H5A	0.2413	0.5936	−0.2498	0.046*	
C6	0.16218 (12)	0.4236 (2)	−0.10814 (10)	0.0295 (4)	
C7	0.10081 (12)	0.3636 (2)	−0.05544 (10)	0.0316 (4)	
H7A	0.0383	0.3721	−0.0626	0.038*	
C8	0.13689 (12)	0.2909 (2)	0.00784 (10)	0.0301 (4)	
C9	0.07088 (12)	0.2201 (3)	0.06351 (11)	0.0345 (4)	
S1	0.0000	−0.14201 (8)	0.2500	0.0295 (2)	
O9	0.07896 (13)	−0.2429 (2)	0.22937 (9)	0.0630 (6)	
O11	−0.02520 (10)	−0.0310 (2)	0.18688 (9)	0.0533 (5)	
O1W	0.0000	0.3439 (3)	0.2500	0.0540 (6)	
H1WA	−0.024 (5)	0.266 (8)	0.286 (3)	0.20 (3)*	

### Atomic displacement parameters (Å^2^)

**Table:**

	*U*^11^	*U*^22^	*U*^33^	*U*^12^	*U*^13^	*U*^23^
O1	0.0398 (8)	0.0540 (9)	0.0318 (7)	0.0075 (6)	−0.0091 (6)	0.0022 (6)
O2	0.0509 (9)	0.0893 (13)	0.0258 (7)	0.0019 (9)	0.0010 (6)	−0.0032 (7)
O3	0.0511 (10)	0.0602 (10)	0.0645 (11)	0.0225 (8)	0.0302 (8)	0.0198 (9)
O4	0.0501 (9)	0.0412 (8)	0.0510 (9)	0.0022 (7)	0.0229 (7)	0.0079 (7)
N1	0.0301 (8)	0.0445 (9)	0.0285 (8)	−0.0057 (7)	0.0029 (6)	0.0030 (6)
N2	0.0331 (8)	0.0436 (9)	0.0306 (8)	−0.0038 (7)	−0.0061 (6)	0.0050 (7)
C1	0.0392 (10)	0.0365 (10)	0.0260 (9)	−0.0010 (8)	−0.0034 (7)	−0.0017 (7)
C2	0.0283 (8)	0.0305 (9)	0.0253 (8)	0.0027 (7)	−0.0014 (6)	−0.0028 (6)
C3	0.0239 (8)	0.0355 (10)	0.0284 (8)	−0.0007 (7)	−0.0010 (6)	−0.0025 (7)
C4	0.0264 (8)	0.0330 (9)	0.0252 (8)	−0.0035 (7)	0.0010 (6)	−0.0035 (7)
C5	0.0404 (10)	0.0445 (11)	0.0295 (9)	−0.0061 (9)	−0.0012 (8)	0.0035 (8)
C6	0.0281 (8)	0.0328 (9)	0.0275 (8)	−0.0020 (7)	−0.0039 (6)	−0.0003 (7)
C7	0.0222 (8)	0.0381 (10)	0.0345 (9)	−0.0011 (7)	0.0002 (7)	0.0012 (7)
C8	0.0292 (8)	0.0321 (9)	0.0291 (9)	0.0009 (7)	0.0040 (7)	−0.0014 (7)
C9	0.0287 (9)	0.0426 (11)	0.0323 (9)	0.0032 (7)	0.0055 (7)	0.0038 (8)
S1	0.0309 (3)	0.0334 (4)	0.0243 (3)	0.000	0.0043 (2)	0.000
O9	0.0814 (13)	0.0702 (12)	0.0374 (8)	0.0449 (10)	0.0205 (8)	0.0090 (8)
O11	0.0300 (7)	0.0781 (11)	0.0517 (9)	0.0056 (8)	0.0054 (6)	0.0308 (8)
O1W	0.0597 (16)	0.0487 (14)	0.0538 (15)	0.000	−0.0195 (11)	0.000

### Geometric parameters (Å, °)

**Table:**

O1—C1	1.319 (2)	C2—C8	1.425 (2)
O1—H28	0.8499	C3—C4	1.389 (3)
O2—C1	1.205 (2)	C3—H3A	0.9300
O3—C9	1.204 (2)	C4—C6	1.395 (3)
O4—C9	1.306 (3)	C5—H5A	0.9300
O4—H21	0.8496	C6—C7	1.389 (3)
N1—C5	1.320 (3)	C7—C8	1.377 (3)
N1—C4	1.388 (2)	C7—H7A	0.9300
N1—H25	0.8747	C8—C9	1.499 (2)
N2—C5	1.325 (3)	S1—O9^i^	1.4498 (16)
N2—C6	1.382 (2)	S1—O9	1.4498 (16)
N2—H22	0.9011	S1—O11^i^	1.4745 (15)
C1—C2	1.493 (2)	S1—O11	1.4745 (15)
C2—C3	1.379 (2)	O1W—H1WA	0.95 (6)
			
C1—O1—H28	103.7	N1—C5—H5A	124.9
C9—O4—H21	113.0	N2—C5—H5A	124.9
C5—N1—C4	108.51 (16)	N2—C6—C7	132.38 (17)
C5—N1—H25	119.9	N2—C6—C4	106.03 (15)
C4—N1—H25	131.5	C7—C6—C4	121.58 (17)
C5—N2—C6	108.92 (16)	C8—C7—C6	116.90 (17)
C5—N2—H22	124.9	C8—C7—H7A	121.6
C6—N2—H22	126.1	C6—C7—H7A	121.6
O2—C1—O1	123.95 (17)	C7—C8—C2	121.67 (16)
O2—C1—C2	122.41 (18)	C7—C8—C9	117.03 (16)
O1—C1—C2	113.55 (16)	C2—C8—C9	121.30 (16)
C3—C2—C8	120.82 (16)	O3—C9—O4	123.86 (18)
C3—C2—C1	119.15 (16)	O3—C9—C8	122.97 (18)
C8—C2—C1	119.28 (16)	O4—C9—C8	113.02 (16)
C2—C3—C4	117.14 (16)	O9^i^—S1—O9	114.29 (17)
C2—C3—H3A	121.4	O9^i^—S1—O11^i^	108.80 (9)
C4—C3—H3A	121.4	O9—S1—O11^i^	108.33 (10)
N1—C4—C3	131.72 (17)	O9^i^—S1—O11	108.33 (10)
N1—C4—C6	106.39 (15)	O9—S1—O11	108.80 (9)
C3—C4—C6	121.81 (16)	O11^i^—S1—O11	108.15 (16)
N1—C5—N2	110.14 (17)		

Symmetry codes: (i) −*x*, *y*, −*z*+1/2.

### Hydrogen-bond geometry (Å, °)

**Table:**

*D*—H···*A*	*D*—H	H···*A*	*D*···*A*	*D*—H···*A*
O1W—H1WA···O3^i^	0.96 (6)	2.26 (5)	2.9012 (11)	123.6
O1W—H1WA···O11^i^	0.96 (6)	2.47 (6)	3.1575 (16)	128.4
O4—H21···O11	0.85	1.79	2.6330 (11)	169
N2—H22···O1W^ii^	0.90	1.96	2.8365 (10)	163
N1—H25···O11^iii^	0.88	1.82	2.6931 (12)	175
O1—H28···O9^iv^	0.85	1.84	2.6616 (11)	161
C5—H5A···O2^v^	0.93	2.20	3.098 (3)	162

Symmetry codes: (i) −*x*, *y*, −*z*+1/2; (ii) −*x*, −*y*+1, −*z*; (iii) *x*+1/2, −*y*+1/2, −*z*; (iv) −*x*+1/2, *y*+1/2, *z*; (v) *x*, −*y*+1, *z*−1/2.
